# Stackelberg Game-Based Power Allocation for V2X Communications

**DOI:** 10.3390/s20010058

**Published:** 2019-12-20

**Authors:** Erqing Zhang, Sixing Yin, Huisheng Ma

**Affiliations:** School of Information and Communication Engineering, Beijing University of Posts and Telecommunications, Beijing 100876, China; yinsixing@bupt.edu.cn (S.Y.); mhs@bupt.edu.cn (H.M.)

**Keywords:** Stackelberg game, power allocation, V2X communications, URLLC, optimization

## Abstract

Ultra-reliable low-latency communication (URLLC) is one of the three usage scenarios anticipated for 5G, which plays an important role in advanced applications of vehicle-to-everything (V2X) communications. In this paper, the Stackelberg game-based power allocation problem was investigated in V2X communications underlaying cellular networks. Assuming that the macro-cellular base station (MBS) sets the interference prices to protect itself from the V2X users (VUEs), the Stackelberg game was adopted to analyze the interaction between MBS and VUEs, where the former acts as a leader and the latter act as followers. For MBS, we aimed at maximizing its utility from interference revenue while considering the cost of interference. Meanwhile, the VUEs aimed at maximizing their utilities per unit power consumption. We analyzed the Stackelberg model and obtained the optimal prices for MBS and optimal transmit powers for VUEs. Simulation results demonstrated the superiority of the proposed Stackelberg game-based power allocation scheme in comparison with the traditional power allocation strategy. Meanwhile, the proposed scheme achieved a better trade-off between economic profit and power consumption.

## 1. Introduction

With the exponential growth of data services and smart terminals, there is a huge demand for wireless communications with higher speed, huger capacity, and quality of service (QoS) guarantee. The emergence of 5G technology will provide strong support for higher wireless capacities in a wide range of applications to meet the requirements in terms of capacity, latency, cost efficiency, and so on. As one of the 5G key technologies, device-to-device (D2D) communications allow user equipment (UE) to directly communicate with other neighboring UEs instead of evolved NodeB [1,2]. As a consequence, D2D communications underlaying cellular communication system can take the advantages of frequency reuse gain and adjacency gain, which dramatically increase the system throughput and network capacity, reduce transmission delay, and save energy [3,4].

Besides, more congested urban roads and frequent traffic accidents make intelligent transportation highly demanded. One of the typical scenarios for D2D communication enhanced by the internet of thing (IoT) is V2X (Vehicle-to-everything) communication in the internet of vehicles. D2D based on terminal direct communication has its inherent advantages in terms of vehicle safety due to its characteristics, such as communication delay and proximity discovery. As one of the three major application scenarios in 5G, URLLC is able to provide V2X with enhanced system capabilities and better coverage. In view of the aforementioned advantages, information on road conditions and the surrounding environment can be effectively shared among vehicles and road infrastructures, which could further facilitate automated driving.

Recently, there have been volumes of existing literature on V2X communications [5,6,7,8,9,10]. In [5], a cooperative automated driving (CAD) system based on collective perception and cooperative maneuver coordination was proposed, which could be applied to several use cases. The authors of [6] investigated the impact of the new radio (NR) flexible numerology on the cellular-vehicle-to-anything (C-V2X) autonomous access mode. The authors of [7] examined the possibility of using the V2X systems at sea and discussed the challenges. In [8,9], the performance comparison of IEEE 802.11p and C-V2X was presented. Moreover, the temporal and spatial dynamics of the V2X network were investigated in [10].

In spite of the above attractive features and application scenarios, there are also a great number of problems in the coexistence of V2X communication and other networks, such as the resource allocation (RA), interference management on account of co-channel interference (CCI) caused by spectrum reuse. In order to solve these problems and achieve technical breakthroughs, several related works have been done in terms of RA, power control, congestion control, link scheduling, interference coordination, and so on [11,12,13,14,15,16,17,18,19,20,21,22,23]. The authors of [11] focused on the existing RA algorithms for V2X communications, and these algorithms were classified and compared with each other according to selected criteria. In [12,13], an RA problem among safety VUEs, non-safety VUEs, and conventional cellular UEs (CUEs) was studied. The authors of [14] proposed a novel hybrid scheme based on C-V2X technology to improve latency and reliability performance. A cooperative solution for V2X communications was proposed in [15], which could guarantee reliability and latency requirements for 5G enhanced V2X services. In [16], the RA problem for D2D-based V2X communication was investigated. In [17,18], the congestion control problem in C-V2X was investigated. The authors of [19,20,21] investigated the radio resource management (RRM) for V2X communications, including both vehicle-to-infrastructure (V2I) and vehicle-to-vehicle (V2V) communication. The author of [22] proposed the joint clustering method and power control scheme in V2X communications to reduce the communication complexity, as well as the total power consumption of base station (BS). Besides, an age of information (AoI)-aware transmission power and resource block (RB) allocation technique has been proposed for vehicular communications [23].

With extensive applications of high-speed multimedia services, there has been a lot of service data to be processed by the terminal equipment. However, the latest battery technology has already been the bottleneck of industrial development, which restrains the development of new technologies in wireless communication. During the vehicle driving process, frequent start-stop or acceleration consumes a large amount of power, especially for pure electric vehicles (EVs) because the battery is its only energy source [24]. In order to increase the mileage of vehicles, power allocation is one of the most critical aspects of vehicle communications. To tackle this issue, researchers from both academia and industry begin to pay more attention to energy efficiency (EE) in V2X communication, and some progress has been made [25,26,27,28,29]. The authors of [25] proposed an energy-efficient relay assisted transmission scheme based on V2X communications in the uplink cellular networks for delay insensitive applications. In [26], an energy-efficient V2X-enabled transmission scheme in uplink cellular networks was proposed aiming to minimize the uplink energy consumption while considering both circuit power and transmit power. The authors of [27] considered the power control problem in V2X networks and proposed a joint beamforming and power control method for downlink V2X communication. In [28], a new power allocation method was proposed, which could dynamically and precisely allocate power to loads. A decentralized power allocation strategy for the EV charging network was proposed in [29].

Game theory is a very important microeconomics tool to analyze the interaction among various agents with different objectives, and there have already been some achievements in RA of V2X communication [30,31,32,33,34,35]. In [30,31], a secure and efficient vehicle to grid (V2G) energy trading framework by exploring the blockchain and edge computing was proposed. The authors of [32] proposed a charging scheme for EVs in a smart community (SC) integrated with renewable energy sources (RES) using a game-theoretical approach. An auction-based channel allocation and power control algorithm for V2V communications was proposed in [33]. In [34], a platoon-assisted vehicular edge computing (PVEC) system was proposed to enhance the efficiency and success of offloading. The authors of [35] investigated parked vehicle edge computing and explored opportunistic resources from parked vehicles (PVs) to run distributed mobile applications. PVs coordinate with VEC servers for collective task execution.

According to the research works mentioned above, it can be seen that there is some literature on RA to maximize the system throughput in a V2X communication system. However, few research works can be found about the interaction effect between the cellular network and V2X communications from the perspective of power-saving. In this paper, the power allocation problem was studied in V2X communication underlaying cellular networks, and interference that the macro-cellular base station (MBS) suffers from V2X users was taken into account. Stackelberg game was adopted to formulate the interaction between MBS and VUEs, and in the Stackelberg game model, MBS acts as the leader while VUEs act as the followers. Then, we presented a joint interference pricing and power allocation scheme aiming at maximizing the utilities of both VUEs and MBS, subject to the average transmission delay constraints of VUEs. Note that the objective of VUEs was the ratio of profit to total consumed power to further achieve power saving.

Since the optimization problem is non-convex in a fraction form, it is very difficult to find the optimal solution. In order to facilitate the computation, the fractional programming can be transformed into an equivalent parameter programming problem according to the Theorem in [36]. Then, through resolving an equivalent problem, the optimal powers for VUEs and interference price for interference can be obtained. In brief, the contribution of this paper can be summarized as follows:

- We investigated the power allocation problem aiming at jointly maximizing the utilities of both the VUEs and MBS.
- By transforming a nonlinear fractional programming problem into equivalent parametric programming, we maximized both the above-mentioned utilities simultaneously.

The organization of this paper is as follows. In Section 2, the system model of the V2X communications underlaying cellular network is described. Section 3 gives the problem formulation, and the Stackelberg game model is analyzed, then a power allocation scheme to achieve a performance tradeoff between economic profit and power consumption is detailed. Simulation results are demonstrated in Section 4, and the whole paper is concluded in Section 5.

## 2. System Model and Problem Formulation

### 2.1. System Model

**Figure 1:**
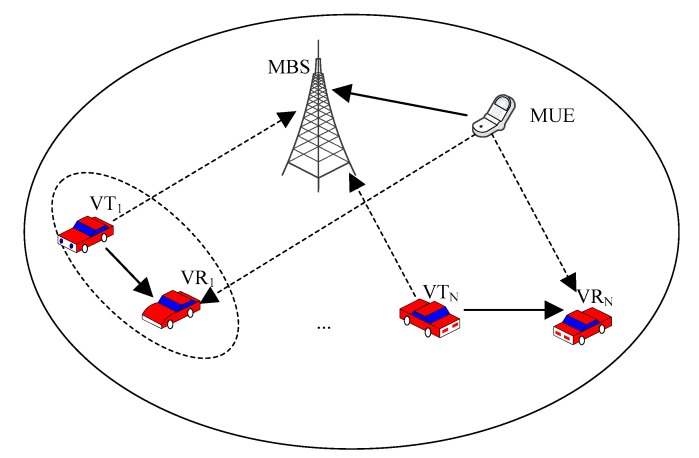
Spectrum sharing model between macrocell and V2X (vehicle-to-everything) communications.

Consider a single-cell scenario where VUEs can exchange information with each other by reusing the available uplink (UL) spectrum resource allocated to CUEs. On the one hand, using the UL cellular spectrum reduces the impact of the interference caused by VUEs at the Evolved Node B (eNB). On the other hand, UL CUEs generate less interference to VUEs since the average transmit power of CUEs is significantly lower than that of a BS. In our scenario, the underlay mode was employed, and broadcasting service was considered, i.e., each vehicle broadcasts its messages to others. It was assumed that the MBS was located in the center, and several macrocell user equipments (MUEs) randomly located. Moreover, some pairs of VUEs, including V2X transmitters (VTs) and corresponding receivers (VRs), were randomly located in the same cell. As shown in Figure 1, the links marked with solid arrows denote the communication links, whereas the interference links are expressed by the dashed arrows.

The distance information between different nodes was considered in our scenario [37,38]. It was assumed that the MBS exactly knew the location [39,40] of CUEs and VUEs present in its cell. In order to guarantee acceptable average interference between CUEs/MBS and VUEs, we assumed that the distance information between different nodes satisfied the following conditions:

- The distance between VTs and the MBS must be large than the distance between the UL CUEs and the MBS.
- The distance between CUEs and the VRs must be large than the distance between the UL CUEs and the MBS.

We considered a macrocell with K UEs and N VUE pairs. In Figure 1, $VT_{i}$ and $VR_{i}$ denote the V2X transmitter i and receiver i, respectively, where i ∈{1, 2, …, N}. It was assumed that all the VUEs were equipped with one single antenna and the channels were block-fading to ensure that the channels remain constant during each transmission block, but possibly change across different blocks. The channel power gain between MUE k and MBS was denoted by $g_{0k}$, and that between $VT_{i}$ and $VR_{i}$ was denoted by $g_{i}$. $h_{i}$ denotes the channel power gain between $VT_{i}$ to MBS, and $l_{ik}$ denotes the channel power gain between MUE k to $VR_{i}$, respectively. All the channel power gains were assumed to be independent and identically distributed (i.i.d.) random variables, and the additive Gaussian white noise with zero mean and variance $\sigma^{2}$ was considered.

In consideration of spectrum sharing between V2X communications and cellular networks, the co-channel interference caused by VUEs would have a significant effect on the performance of the cellular networks. In order to effectively protect the communication quality of cellular networks, interference pricing was introduced.

Under the above underlying framework, considering that MUE transmits at a certain power $P_{P}$ and $VT_{i}$ transmits with variable power $P_{i}$, the received signal to interference and noise ratio (SINR) at $VR_{i}$ can be expressed as follows.

(1) $$\gamma_{i}\left(P_{i}\right)=\frac{P_{i}g_{i}}{\sum_{k=1}^{K}P_{P}l_{ik}+\sigma^{2}}$$

In this paper, the profit per Joule was considered in V2X communications for improving the energy utilization. The aim of $VT_{i}$ was to maximize its own utility from the perspective of power-saving while not affecting the normal transmission of cellular network, which could be given as follows:

(2) $$\begin{array}{c} \begin{array}{c} \eta_{i}\left(P_{i},p_{i}\right)=\frac{\xi_{i}\;log_{2}\left(1+\gamma_{i}\left(P_{i}\right)\right)-p_{i}I_{i}\left(P_{i}\right)}{P_{c}+P_{i}} \end{array} \end{array}$$

where $\xi_{i}$ is a conversion factor to denote the economic gain of VUE i from its per unit of rate, $p_{i}$ denotes the price determined by MBS, $I_{i}(P_{i})$ denotes the interference that $VT_{i}$ intends to buy from the MBS under the interference price $p_{i}$ with $I_{i}\left(P_{i}\right)≜P_{i}h_{i}$, and $P_{c}$ denotes the additional circuit power consumption of devices during transmission, which is independent to the data transmission power.

### 2.2. Problem Formulation

In this section, we presented the Stackelberg game model for the price-based power allocation problem in V2X communications. The Stackelberg model is a strategic game in economics in which the leader moves first and then the follower moves sequentially. In the game theory terms, the players of this game are composed of a leader and multiple followers, and the leader is sometimes referred to as the market leader. The followers can take action based on the observed action of the leader.

Considering the cellular network and V2X communications mentioned above, we formulated a power allocation problem in the heterogeneous network as a two-stage Stackelberg game, which consisted of one leader and N followers. The MBS was regarded as the leader who acts first, then VUEs were followers who that act subsequently by observing the leaders’ action (strategies). More specifically, the MBS did set the interference power price (denoted by $p_{i}$) for VUE i initially, then the $VT_{i}$ decided its own transmission power $P_{i}$ according to the interference power price $p_{i}$. Therefore, the two-stage Stackelberg game model was formulated as follows. 

Based on the above analysis, the objective of MBS was to maximize its utility by means of imposing a pecuniary charge for the interference caused by VUEs while considering the effect of interference on the utility, which could be mathematically written as

(3) $$\begin{array}{c} \begin{array}{c} \begin{array}{c} U_{MBS}\left(p_{i},P\right)=\sum_{i=1}^{N}\left\{p_{i}I_{i}\left(P_{i}\right)-\alpha I_{i}\left(P_{i}\right)\right\} \end{array} \end{array} \end{array}$$

where α is another conversion factor to denote the MBS’s economic loss from per unit of interference caused by VUEs, $I_{i}(P_{i})$ denotes the interference from $VT_{i}$, and P denotes the vector of power allocation for all V2X users, i.e., $P={\left[P_{1},P_{2},\cdots ,P_{N}\right]}^{T}$.

It is noteworthy that the $P_{i}$ was actually a function of price $p_{i}$ in this Stackelberg game model (i = 1, 2, … , N), indicating that the power allocated to VUEs was completely dependent on the interference price $p_{i}$. Therefore, MBS had to find the optimal interference price $p_{i}$ so as to obtain its maximal utility. From the problems discussed above, it should be clear that the optimal $p_{i}$ could be obtained by solving the following optimization problem:

(4) $$OP1:\;\underset{p\geq 0}{\max}\;U_{MBS}\left(p,P\right)$$

where p denotes the vector of interference price with $p={\left[p_{1},p_{2},\cdots ,p_{N}\right]}^{T}$, and $p_{i}$ is the interference price of V2X transmitter i.

For V2X communication, the profit per unit of energy was considered as the utilities of VUEs. According to Equation (2), let $U_{i}\left(P_{i},p_{i}\right)=\eta_{i}\left(P_{i},p_{i}\right)$, then we found that the utility of $VT_{i}$ consisted of two parts: revenue per Joule and cost per Joule. On the one hand, $VT_{i}$ decided to increase its transmit power, and the transmit data rate would increase accordingly, improving its transmission quality. On the other hand, the increased transmit power would cause more severe interference to MBS, and $VT_{i}$ had to pay more for it, leading to higher cost incorporated into the overall utility. Therefore, it was necessary to find the optimal allocated power for $VT_{i}$, which motivated us to develop a power allocation scheme with the objective aiming at maximizing the utility of $VT_{i}$. In addition, we assumed that the data buffers of VTs were extremely large, where the data packets were always waiting to transmit while data buffers of VTs were limited, and the arrival process of packets could be modeled as a Poisson process. To guarantee the QoS requirements of VUEs, the requirement for low average transmission delay should be satisfied. The problem mentioned above of $VT_{i}$ could be described mathematically by another optimization problem:

(5) $$\begin{array}{lll} OP2: & \underset{P_{i}\geq 0}{\max} & U_{i}\left(P_{i},p\right)\\ & s.t. & E\left[W_{i}\right]\leq T_{i} \end{array}$$

where $W_{i}$ denotes the time that the packets of the VT i wait in the queue plus the service time whose expectation can be denoted by $E\left[W_{i}\right]$. $T_{i}$ denotes the delay constraint in terms of time. Since the delay constraint condition is intractable, we transformed the delay constraint into a transmission rate constraint of the VUE i by modeling the data buffer of VUE as an M/G/1 queue [41], which is given by:

(6) $$R_{i}\geq \varphi \left(T_{i},\upsilon_{i},Z\right)$$

where $R_{i}=\log_{2}\left(1+\gamma_{i}\left(P_{i}\right)\right)$, $\upsilon_{i}$ denotes the independent packet arrival rate of the VUE i, which can be modeled as a Poisson process, and Z represents the packet size [42], which is a random variable.

According to Equation (5), V2X transmitters will compete with each other through the non-cooperative game to maximize their utilities, which could be written as follows:

(7) $$\begin{array}{c} \begin{array}{c} \begin{array}{c} \begin{array}{c} \begin{array}{c} \begin{array}{c} \begin{array}{c} G=\{\Omega ,{\left\{P_{i}\right\}}_{i\in \Omega },\left\{U_{i}\left(P_{i},p\right){\}}_{i\in \Omega }\right\} \end{array} \end{array} \end{array} \end{array} \end{array} \end{array} \end{array}$$

where Ω={1,2,⋯,N} denotes the set of VTs, $P_{i}$ denotes the power of VT i.

The above two optimization problems together formed the Stackelberg game model, and our aim was to find the Stackelberg equilibrium (SE) point(s), which have been elaborately detailed in the following parts.

## 3. Proposed Algorithm

Firstly, the Stackelberg equilibrium (SE) was defined for the proposed game, and then the fractional structure of the VUEs’ objective function was analyzed and subsequently transformed into an equivalent parametric programming problem. Finally, the power allocation scheme for V2X communications was deduced.

### 3.1. Stackelberg Equilibrium

For the Stackelberg game abovementioned, it is worth noting that for VUEs, P is actually a function with respect to p; in other words, the power of VUE i depends on the interference power price $p_{i}$ decided by MBS. The SE is defined as:

**Definition** **1.** *Let*$p^{*}$*be the optimal solution of*OP1,*and*$P^{*}$*be the optimal solution for the VUEs in*OP2*, then the point (*$p^{*}$, ${\;P}^{*}$*) is a SE for this proposed Stackelberg game if, for any*(p,P)*, the following conditions can be satisfied:*

(8) $$U_{MBS}\left(p^{*},P^{*}\right)\geq U_{MBS}\left(p,P^{*}\right)$$

*and*

(9) $$U_{i}\left(P_{i}^{*},p^{*}\right)\geq U_{i}\left(P_{i},p^{*}\right)$$

*for*∀i*with*p≥0*and*P≥0.

Based on the analysis above, it is clear that solving this Stackelberg game problem for its SE is equivalent to finding its sub-game perfect Nash equilibrium (NE), where VUEs compete with each other in a non-cooperative manner, then a non-cooperative power allocation sub-game is formulated at VUE side. For MBS, the optimal interference price can be obtained by solving the problem OP1, which should be based on the power of VUEs, since the MBS derives its optimal strategy ($p^{*}$) according to that (P) of V2X communications. Then, the VUEs update their powers to the optimal power ($P^{*}$) derived through resolving the problem OP2 based on $p^{*}$, which will converge to a stable condition.

### 3.2. Mathematical Analysis

It is noteworthy that the model of the V2X transmitter i is a non-linear fractional programming problem, which is very hard to solve directly. In order to facilitate the computation, an equivalent non-linear parametric programming model was deduced from the original problem, and the mathematical description of the theory could be briefly given as follows.

There is a fractional programming problem as follows:

(10) $$\underset{x}{\max}\;q=\frac{N\left(x\right)}{D\left(x\right)}$$

Another one is the parametric programming problem:

(11) $$\underset{x}{\max}\;N\left(x\right)-qD\left(x\right)$$

Let F(q) = max{N(x) − qD(x)}, where q is a key parameter. Therefore, F(q) is a convex function of q.

Let $x^{*}$ be the solution of Equation (11), that is, $q^{*}=N\left(x^{*}\right)/D\left(x^{*}\right)$, then the sufficient and necessary conditions q = q^∗^ is F(q) = 0. Thus, searching for the optimum of problem Equation (11) is equal to find the zero root of F(q) = 0.

In our model, the non-linear parametric programming problem equivalently transformed from the original fractional programming problem OP2 of the V2X transmitter i could be given as follows:

(12) $$\begin{array}{lll} OP3: & \underset{P_{i}\geq 0}{\max} & U_{ti}\left(P_{i},p\right)\\ & s.t. & R_{i}\geq R_{th} \end{array}$$

where $R_{th}=\varphi \left(T_{i},\upsilon_{i},Z\right)$ and

(13) $$U_{ti}\left(P_{i},p_{i}\right)=\xi_{i}\log_{2}\left(1+\gamma_{i}(P_{i})\right)-p_{i}I_{i}(P_{i})-q_{i}(P_{c}+P_{i})$$

Then, the optional solution of OP2 could be transformed to find the zero root of the problem OP3.

### 3.3. Power Allocation Scheme

In this section, we analyzed the Stackelberg game in detail and derived the optimal solution of the game model in our scenario. On the basis of the analysis above, the existence and uniqueness of the Stackelberg game were proved, respectively. Furthermore, the update function of the interference power price for VUEs was given, and it could converge to the unique equilibrium. Finally, the power allocation scheme was proposed.

In the proposed Stackelberg game, the leader acted first, and the followers, subsequently took corresponding actions, that is, the MBS did set the interference prices first, and then the VTs decided their optimal transmit powers according to the given interference price. It is noteworthy that the action of the leader (MBS) could be observed by VUEs, and then the problem could be solved with the backward induction method.

#### 3.3.1. Analysis of VUEs-Level Game

**Theorem** **1.** *For any given interference price* p *by MBS, there exists a unique Nash equilibrium in a non-cooperative game for VUEs.*

**Proof** **.** Please refer to Appendix A.□

Assuming that interference price $p_{i}$ was fixed by MBS in the proposed game, and the q was a constant, then the best transmit power of VUEs could be derived by solving

(14) $$\frac{\partial U_{ti}}{\partial P_{i}}=\frac{\xi_{i}}{\ln2}\frac{g_{i}}{G_{i}+P_{i}g_{i}}-p_{i}h_{i}-q_{i}=0$$

where $G_{i}=P_{P}l_{i}+\sigma^{2}$ and $U_{ti}$ denotes the $U_{ti}\left(P_{i},p_{i}\right)$, respectively. Then, the closed-form solution of optimal transmit power could be expressed as follows:

(15) $$P_{i}^{*}=\frac{\xi_{i}}{\ln2\left(p_{i}h_{i}+q_{i}\right)}-\frac{G_{i}}{g_{i}}$$

#### 3.3.2. Analysis of MBS-Level Game

Substituting the Equation (15) into (4), the utility of MBS could be rewritten as:

(16) $$\begin{array}{c} \begin{array}{ccc} \max\;\;U_{MBS} & = & \overset{N}{\underset{i=1}{\sum }}p_{i}P_{i}h_{i}-\overset{N}{\underset{i=1}{\sum }}\alpha P_{i}h_{i}\\ & = & \overset{N}{\underset{i=1}{\sum }}\left(p_{i}-\alpha \right)P_{i}h_{i} \end{array} \end{array}$$

It could be found out from the Equation (16) that there existed a tradeoff between prices and the utility of MBS. For instance, the MBS firstly did set a low interference price $p_{i}$, then the VUEs sharing the same spectrum would transmit with high power, leading to an increase of the MBS’s utility as $p_{i}$ rises based on Equation (16). However, with a further increase in the interference price $p_{i}$, the growth of the MBS’s utility would be obviously restrained. In that case, the transmit power $P_{i}$ of VUE i would decrease with the increasing of $p_{i}$, resulting in the reduction of the utility of MBS. Therefore, there existed the best choice for an interference price $p_{i}$ corresponding to the optimal utility for MBS.

Hence, the optimal interference price $p_{i}$ could be obtained by solving the following equation:

(17) $$\frac{\partial U_{MBS}}{\partial p_{i}}=\left(p_{i}h_{i}-\alpha h_{i}\right)\frac{\partial P_{i}}{\partial p_{i}}+P_{i}h_{i}=0$$

Based on the above-mentioned analysis, the optimal interference price $p_{i}$ was the function of the $G_{i}$, which could be expressed as follows.

(18) $$p_{i}^{*}=p_{i}^{*}\left(G_{i}\right)$$

Equation (17) could be simplified and transformed into:

(19) $$p_{i}=Z_{i}\left(p_{i}\right)=-\frac{P_{i}}{\partial P_{i}/\partial p_{i}}+\alpha$$

In order to obtain the $p_{i}$ in Equation (19), the MBS needed to know the exact and prompt feedback information regarding the $P_{i}$ and $\partial P_{i}/\partial p_{i}$ from the VUEs. Then, the interference price p of MBS could be updated according to the formula below:

(20) p=Z(p)

where $Z(p)={\left[Z(p_{1}),Z(p_{2}),\cdots ,Z(p_{N})\right]}^{T}$, which represents interference price competition constraint for MBS. Therefore, the update process of price p could be modeled by an iterative formula as follows:

(21) p(t+1)=Z(p(t))

Based on the Stackelberg game analysis and the price updating formula, the MBS could obtain the optimal interference price $p_{i}^{*}$ for VUEs, and then the VUEs would transmit with the optimal power $P_{i}^{*}$, which would actualize their maximum utilities.

It is noteworthy that the aforementioned mathematic deduction was based on the assumption that the parameter q was constant. However, according to the F(q) = 0 in Equation (11), q in OP 3 was a variable needed to be solved. Considering that the function F(q) was monotonically decreasing with respect to q, the bisection search could be utilized to find the optimal solution q^∗^. In summary, the proposed power allocation algorithm is detailed in Algorithm 1.
**Algorithm 1: Power Allocation Scheme****Require:**  **g**, **h**, **l**, **ξ**, N, σ, P_C_, P_P_, α, δ, a and b (error limitation δ > 0, a, b satisfying F (a) > 0 and F (b) < 0); q = (a + b)/2;**Ensure:**  1: while |F (q)|> δ do  2:   **Calculate optimal price and power**  3: Use Equations (15) and (19), calculate the optimal $p^{*}$ and $P^{*}$;  4: k = 1;  5: while |***p***(k) − ***p***(k − 1)| > δ or |***P*** (k) − ***P*** (k − 1)| > δ do  6:   update ***p***(k) and ***P***(k) according to Equations (15) and (19) under the constraints;  7:   k = k + 1;  8:   end while  9: if F (a) · F (q)≥0 then  10: a = q;  11: else  12:   b = q;  13: endif  14:   q = (a + b)/2;  15:   endwhile

## 4. Numerical Results

In this section, numerical results are presented to evaluate the performance of the proposed power allocation scheme in V2X communication on the basis of the interference pricing approach. We considered a cellular network with two MUEs and two VUEs (two VTs and the corresponding VRs) in order to ease the computation. It was assumed that the channel power gains **g**, **h**, and **l** were Rayleigh distributed random variables with mean –6 dB, and the noise variance was 0.05. 

**Figure 2:**
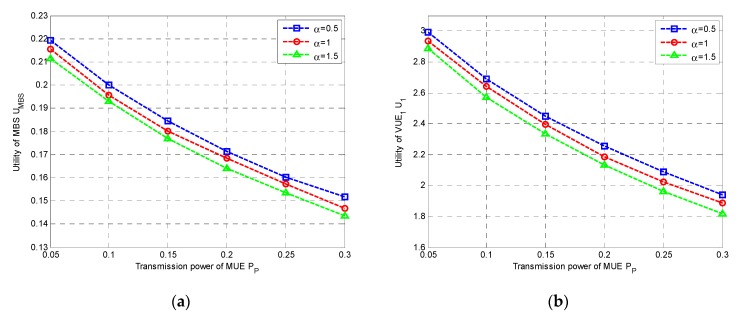
Utilities under different α vs. P_P_: (**a**) Utility of MBS; (**b**) Utility of VT_1_. MBS: macro-cellular base station; VT: V2X transmitter.

**Figure 3:**
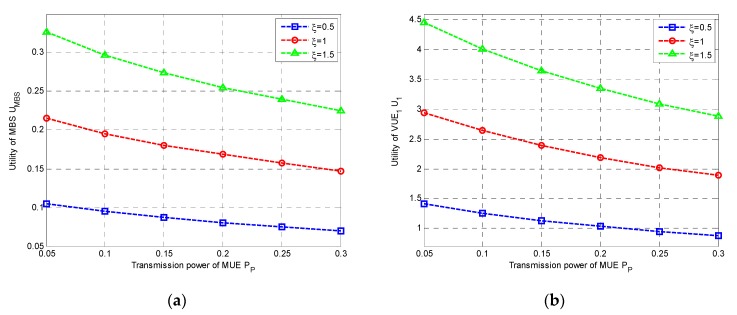
Utilities under different ξ vs. P_P_: (**a**) Utility of MBS; (**b**) Utility of VT_1_.

Firstly, we studied the utilities of both MBS and VUEs. In Figure 2, we plotted the utilities of MBS and VUE_1_ with transmit power of MUE P_P_ under different α, respectively, where α is defined in Equation (3). It was clearly observed that both the utilities were decreased with the increase of the P_P_, since higher P_P_ would cause higher interference to VUEs, leading to performance degradation, in both MBS and VUEs. As shown in Figure 2a,b, the utilities of MBS and VUEs decreased with the increment of α, respectively. However, both the utilities would increase with the increase of ξ in Figure 3a,b. This was probably because that higher weight was assigned to interference suffered by MBS when α gets larger, resulting in the lower utility of MBS. On the contrary, the throughput tended to be more important in the utility of VUE for large ξ, and then a higher utility could be obtained.

**Figure 4:**
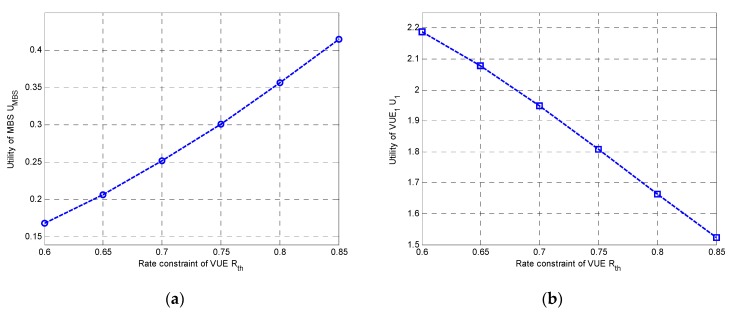
Utilities vs. R_th_: (**a**) Utility of MBS; (**b**) Utility of VT_1_.

Secondly, we studied the utilities of both MBS and VUEs under different rate constraints. In Figure 4a,b, we plotted the utilities of MBS and VUE_1_ with different $R_{th}$, respectively. It could be observed that the utility of MBS was increased with the increase of the $R_{th}$, while the utility of VUE_1_ was decreased with the increase of the $R_{th}$. This was probably because higher $R_{th}$ would cause higher power consumption for VUEs, resulting in higher revenue for MBS. 

**Figure 5:**
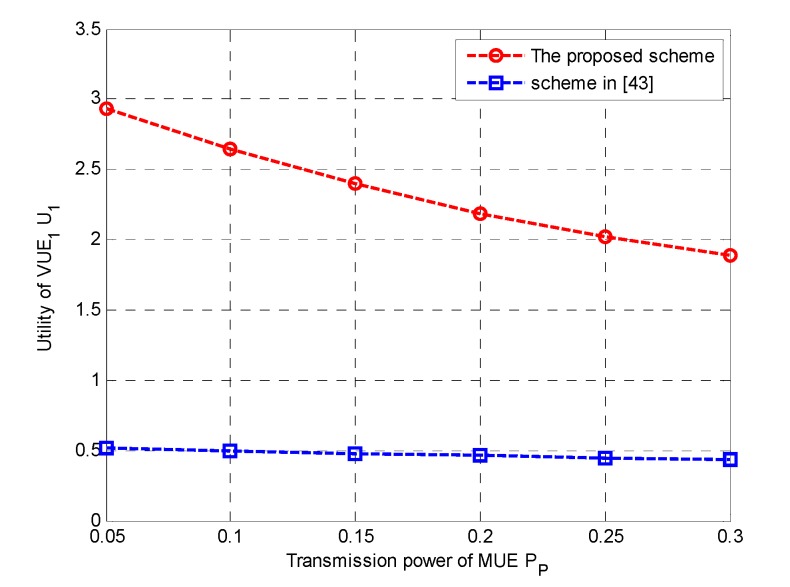
Comparison between the proposed and the traditional utility maximization schemes vs. P_P._

Finally, we evaluated the performance of the proposed power allocation scheme; we presented the simulation results of VUE’s utility with different P_P_. Figure 5 compares the utility of the proposed power allocation scheme and the traditional utility maximization scheme [43] without considering the power consumption. It could be found that the proposed power allocation scheme significantly outperformed the traditional scheme in the aspect of power-saving. The results showed that the proposed scheme could efficiently strike a balance between economic profit and power consumption.

## 5. Conclusions

In this paper, we proposed a power allocation scheme in V2X communications underlying uplink cellular network, for the purpose of maximizing the utilities of MBS and VUEs. We formulated the problem as a bi-objectives optimization model based on the Stackelberg game. The objective function of VUEs could be transformed into an equivalent parametric programming model. The whole optimization problem could be solved by an iterative computational method with a bisection search. Different from conventional strategies, the proposed scheme achieved optimal utility in the view of power-saving, significantly improved the performance of V2X communications, and ultimately attained the Stackelberg equilibrium. Simulation results demonstrated the proposed scheme could obtain a better trade-off between economic profit and power-saving. Further study might focus on location-based resource allocation and performance testing with realistic vehicle traffic flows.

## Appendix A

According to the game theory for wireless engineers, it could be found that if a game model satisfies both of the following criteria, there exists a Nash equilibrium.

1. Strategy space {P_i_} is a non-empty and bounded compact convex subset of Euclidean space;

2. The utility function is a kind of quasi-concave (quasi-convex) function with respect to {P_i_}.

It is obvious that the strategy space {P_i_} meets the first condition for the existence of Nash equilibrium because {P_i_} is a non-empty, bounded, and closed set, as well as a compact convex set.

The first-order partial derivative of U_ti_ with respect to P_i_ can be found in Equation (14), and the corresponding second-order partial derivative of U_ti_ is as follows:

(A1) $$\frac{\partial^{2}U_{ti}}{\partial P_{i}^{2}}=-\frac{\xi_{i}}{\ln2}\frac{g_{i}^{2}}{{\left(G_{i}+P_{i}g_{i}\right)}^{2}}<0$$

Therefore, U_ti_ is a kind of quasi-concave function with respect to {P_i_}, and then the second condition of Nash equilibrium is satisfied.
